# Medium- and Long-Term Re-Treatment of Root Canals Filled with a Calcium Silicate-Based Sealer: An Experimental Ex Vivo Study

**DOI:** 10.3390/ma15103501

**Published:** 2022-05-13

**Authors:** Giulia Bardini, Elisabetta Cotti, Terenzio Congiu, Claudia Caria, Davide Aru, Montse Mercadè

**Affiliations:** 1 Department of Conservative Dentistry and Endodontics, University of Cagliari, 09124 Cagliari, Italy; cottiendo@gmail.com (E.C.); claudiacaria90@icloud.com (C.C.); davide.aru34@gmail.com (D.A.); 2 Department of Medical Science and Public Health, University of Cagliari, 09124 Cagliari, Italy; terenzio.congiu@unica.it; 3 Department of Dentistry, Researcher IDIBELL Institute, 08106 Barcelona, Spain; montsemercade@ub.edu

**Keywords:** bioroot RCS, AH Plus, calcium silicate-based-sealer, epoxy resin-based-sealer, re-treatability

## Abstract

This study investigated the possibility of re-treating a calcium silicate-based sealer (CSBS), compared to an epoxy-resin sealer (RBS), using rotary instrumentation at different times from obturation (1 month/1 year). Thirty-six human mandibular premolars, extracted as a result of orthodontic or periodontal problems, were instrumented and randomly divided into three groups of 12: BR and BR*, which were filled with CSBS and re-treated after one month and one year of storage, respectively, and AH, which was filled with RBS and re-treated after one month. The same re-treatment protocol was used for all teeth, and the times required for the procedure was recorded. The re-treated specimens were longitudinally sectioned and examined at the stereomicroscope (SM) at 20× magnification. Image J Software was used to process the microphotographs. The percentage of residual filling materials in the root canal and the apical third, the ability to reach working length WL and patency, and the time taken to complete the re-treatment were recorded and analyzed by ANOVA and post hoc Bonferroni test (*p* = 0.05). Scanning electron microscopy (SEM) and coupled energy-dispersive spectroscopy (EDS) were applied to representative samples to evaluate canal cleanliness and chemical elements. Patency and WL were re-established in all of the teeth. Residual filling materials were retained in all specimens of the three groups. The mean percentage of residual materials was significantly different between BR and BR* (*p*-value = 0.048), with BR* showing the highest values. The mean time to complete re-treatment was significantly lower for AH, followed by BR (*p* = 0.0001) and BR* (*p* = 0.0078). Conclusions: After both medium and long storage periods, the CSBS can be concluded to have been successfully removed from canals with simple anatomy.

## 1. Introduction

Re-treatments have assumed greater importance in clinical practice since several scientific studies indicated secondary orthograde endodontic therapy to be the treatment of choice for apical periodontitis (AP) as a first-step alternative to retrograde treatment [1,2].

Failure of primary endodontic treatment is most frequently due to the persistence of bacteria and viruses within un-instrumented volumes of the root canal system [3]; thus, accessing every area of the latter becomes an essential requirement for the success of secondary treatments [4].

State-of-the-art endodontic obturation, obtained using gutta-percha (GP) cones in conjunction with a sealer adapted to the canal walls, should prevent microorganisms and fluids from leaking to the periapical tissues by sealing the entire system [5]. Sealers are designed to improve the seal provided by the core obturation material. Currently, many different sealers are being used in endodontics. They can be categorized according to their chemical composition into zinc oxide and eugenol-, calcium hydroxide-, non-eugenol-, glass ionomer-, resin-, silicone-, and calcium silicate-based sealers (CSBS) [6]. Resin-based sealers (RBS) have a long history of use, provide adhesion, and do not contain eugenol. There are two major categories: epoxy resin-based and methacrylate resin-based sealers. Ah Plus (Dentsply International Inc., York, PA, USA) is an epoxy resin-amine-based system in two tubes. The epoxide paste tube contains a diepoxide and fillers as the primary ingredients; the amine paste tube contains a primary monoamine, a secondary diamine, a disecondary diamine, silicone oil, and fillers as the major ingredients. Ah Plus was formulated to avoid the formation of formaldehyde [7]. Hydraulic calcium silicate-based materials were first introduced as root repair cement [8,9] and later as root canal sealer [10]. Calcium silicate materials may include alumina and zirconia, calcium silicates, hydroxyapatite, and calcium phosphates in their formulation [11]. A new CSBS is BioRoot^TM^ RCS, [BR] (Septodont, Saint-Maur-des-Fossés, France), consisting of powder and liquid. According to the manufacturer, the powder mainly consists of tricalcium silicate, povidone, and zirconium dioxide. The liquid is an aqueous calcium chloride solution with polycarboxylate. Alkalizing activity associated with releasing calcium ions, bioactivity with the apatite-forming ability, hard tissue formation, and dentinal tubule penetration was reported for BioRoot RCS [11,12,13].

The interaction of CSBS with the dentinal surface of the canal walls, which produces the formation of a *mineral infiltration zone* [14,15], has led many clinicians to assume that these materials were not easily removable from the canals. Currently, information regarding the re-treatability of CSBS is unclear.

The purpose of this ex vivo study was to assess the removal of the filling material from root canals obturated with either a bioactive hydraulic sealer or an epoxy-resin sealer by evaluating the feasibility to achieve apical patency and the time necessary to complete the procedure and to assess the amount residual sealer within the root canals. The null hypotheses were that there would be no differences in the amount of residual filling materials or the times required for the re-treatment procedure among the groups.

## 2. Materials and Methods

### 2.1. Materials

The experimental study was performed ex vivo and compared two obturation techniques performed by two calibrated, expert operators.

The root canals were obturated either with a novel CSBS (BioRoot^TM^ RCS, [BR]) and the single cone GP technique or with an RBS (AH Plus, [AH]) performed with the warm vertical compaction of the gutta-percha [16].

BioRoot^TM^ RCS is a tricalcium silicate-based bioactive sealer developed to be used in conjunction with GP points and the single cone technique, or with cold lateral condensation of GP, for permanent root canal obturation [17,18].

AH Plus is an epoxy resin-amine-based system traditionally used with a GP master cone, which may be further adapted to the canal using a compaction technique with multiple accessory cones or with heat and a continuous wave of condensation technique [16].

### 2.2. Sample Size Calculation

Sample size calculation was performed using the G*Power v. 3.1.9.4 software (Heinrich Heine, Universität Dusseldorf, Germany). The power analysis indicated that 36 specimens (*n* = 12) were required. This gave at least 80% power to detect a maximum difference between group means. The advisable specimen size was determined based on a previous study that used a similar methodology [19].

### 2.3. Sample Selection

**Figure 1:**
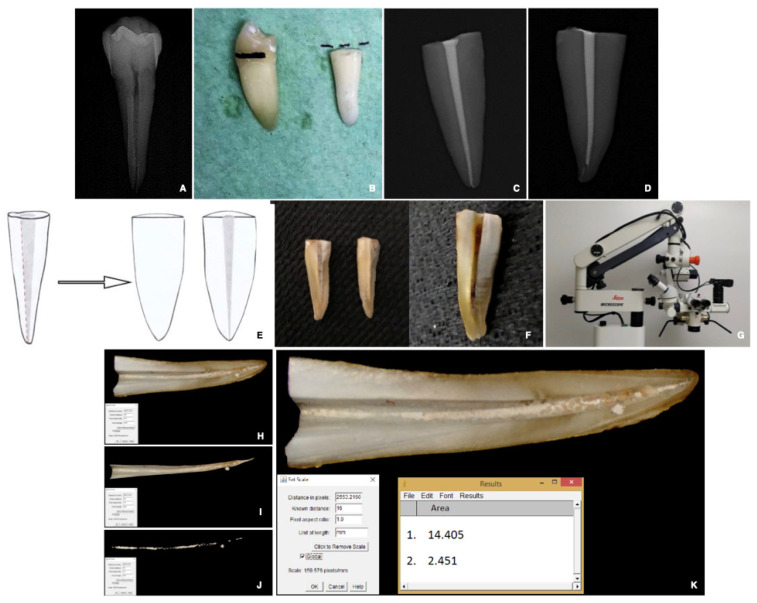
(**A**) A representative specimen of the experimental group. (**B**) The crowns of selected teeth were removed to obtain standardized samples with 16 mm root length. (**C**,**D**) A representative radiograph of two roots obturated at WL (groups) and WL-1 (subgroups), respectively. (**E**,**F**) Teeth were sectioned longitudinally along the main axis, and photographs of each half-root were taken under 20× magnification using a stereomicroscope and a digital camera (Alpha NEX-5; Sony, Tokyo, Japan). (**G**) Leica M655 (Leica Microsystems, Wetzlar, Germany). (**H**–**K**) Image J software (version 1.41, National Institute of Health, Bethesda, MD, USA). An examiner highlighted, for each tooth, the following: root canal surface (**I**), apical third surface, amount of residual root filling materials in the canals, and (**J**) in the apical third. Image J software was used to express the highlighted areas in pixels (**K**).

Thirty-six single-rooted mature, human mandibular premolars extracted for orthodontic or periodontal reasons, with a single straight and regular canal and a completely formed root, were selected. Exclusion criteria were root caries, internal calcifications, and external and internal resorption. Internal resorption was verified with periapical radiographs (Figure 1A). 

### 2.4. Study Procedure

#### 2.4.1. Specimen and Root Canal Preparation

The crowns of the selected teeth were removed with a diamond bur to standardize the specimens at 16 mm root length (Figure 1B).

A #10 K-file (Dentsply Maillefer, Ballaigues, Switzerland) was used to determine the working length (WL) until visible at the apical foramen. Instrumentation of teeth was performed with ProTaper Next Rotary System (Dentsply Maillefer, Ballaigues, Switzerland) to size 30/0.07 taper. A #10 K-file was used to reconfirm patency; each canal was irrigated with 2 mL of 5.25% sodium hypochlorite (NaOCl) solution, followed by 2 mL of sterile saline, and then dried with paper points. 

#### 2.4.2. Root Canal Obturation

The specimens were numbered and randomly divided into three groups (*n* = 12) depending on the material used for root canal filling and the time of re-treatment:

- BR: single-cone GP and BioRoot^TM^ RCS, re-treated after 1 month;
- AH: warm vertical condensation of GP and AH Plus re-treated after one month;
- BR*: single-cone GP and BioRoot^TM^ RCS, re-treated after 12 months.

Two subgroups (*n* = 6) were further created for each group based on the working length chosen for the filling:

In six specimens, the GP cone (master cone) was seated with a tug back at the working length (WL), and in six samples, the cone (pilot cone), not exhibiting a tug back, was placed 1 mm coronal to WL (WL-1), to permit the CSBS to seal the apex.

All canals were filled with a matched-taper single-cone technique to maintain consistency. Each canal was trial fitted with a size 30.06 taper gutta-percha. 

According to the manufacturer’s instructions, both sealers were mixed and placed into the root canals using a coated paper point.

The GP cones were seared at the orifices with a heat carrier (System B, KerrHawe SA, Bioggio, Switzerland), and the accesses were sealed with bonded composite resin.

The specimens were then radiographed to confirm a homogenous root canal filling (Figure 1C,D), and each element was preserved in saline solution at 37 °C.

#### 2.4.3. Re-Treatment Procedure

Following the manufacturer’s instructions, the root filling materials were removed from the canals using ProTaper Universal D1, D2, and D3 files (Dentsply Maillefer, Ballaigues, Switzerland). 

A ProTaper Universal D1 file was used in the coronal third of the canal, the canals were then irrigated with 2 mL of 5.25% NaOCl, ProTaper D2, and D3 files were then used, respectively, to remove the obturation material from the middle and apical third of the canal. Each rotary instrument was used with slight apical pressure and inspected upon each removal. The re-treatment procedures were completed with a #30 k-file and a solvent (Endosolv, Septodont, Saint-Maur-des-Fossés, France) left in the canal for one minute, mostly to remove residual from gutta-percha, and followed by a final rinse with 5.25%. NaOCl.

Throughout the procedure, canal patency was confirmed by manual K-file # 10.

Re-treatment was considered completed when no residual was observed on canal instruments, using a magnifying system (Zeiss 4.5 × 350), and when the canal appeared clear on the intraoral periapical radiograph.

A digital chronometer recorded the times required for the re-treatment procedure in minutes (ADANAC 3000, Marathon, La Chaux-de-Fonds, Switzerland).

The timer was started when an instrument was introduced in the canal and turned off when it was removed. The irrigation time and the time required for changing rotary instruments were not recorded. 

#### 2.4.4. Preparation and Evaluation of the Re-Treated Samples

After re-treatment, the roots were split longitudinally and subsequently photographed (Figure 1E). Grooves were made in a mesiodistal direction along the main axis of each tooth, under constant water cooling, to proceed with a cut in the para-median position of each specimen. The samples were divided into two halves, and the portion containing the complete half-canal was then polished with an Arkansas stone under water cooling (Figure 1F). 

A photograph of each half-root was taken under 20× magnification using a stereomicroscope (SM) (Leica M655, Leica Microsystems, Wetzlar, Germany) and a digital camera (Alpha NEX-5; Sony, Tokyo, Japan) (Figure 1G). 

The images were imported into imaging software (Image J version 1.41; NIH, Bethesda, MD, USA) (Figure 1H–K) and randomly evaluated by a third independent trained examiner. Considering the standardized length of the roots, the scale set associated the image pixels with the corresponding millimeters of the canal. Each canal was isolated from the rest of the tooth to calculate the area of the entire wall (Figure 1H) and its apical 3rd. The *Versatile Wand Tool* plug-in highlighted the area of the whole root canal wall (Figure 1H) and its apical 3rd, and the residual filling materials (RFM) (Figure 1J). RFM was extrapolated and measured (Figure 1K).

Subsequently, the amount of residual filling material (RFM)**,** concerning the area of the canal and of the apical 3rd, was calculated in percentage values by using a proportion [19] as follows:

$$\mathrm{RFM}\;\left(\%\right)=\frac{residual\;filling\;materials\;\;throughout\;the\;area\;of\;the\;entire\;canal\;\left(\mathrm{mm}2\right)}{extension\;of\;the\;entire\;root\;canal\;surface\;\left(mm2\right)}\times 100\;\mathrm{apical}\;3\mathrm{rd}\;\mathrm{RFM}\;\left(\%\right)=\frac{residual\;filling\;materials\;throughout\;the\;area\;of\;the\;apical\;3rd\;of\;the\;canal\;\left(\mathrm{mm}2\right)}{residual\;filling\;materials\;throughout\;the\;area\;of\;the\;entire\;canal\;\left(\mathrm{mm}2\right)}\times 100$$

**Figure 2:**
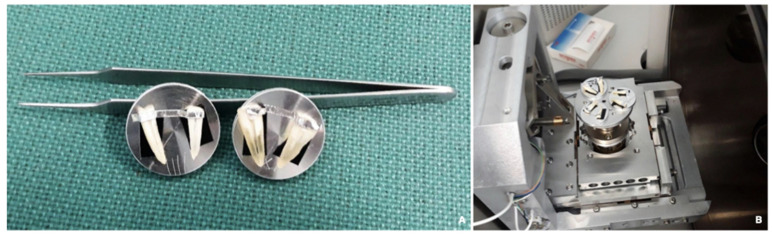
(**A**,**B**) The samples were examined with a scanning electron microscope (SEM), FEI Quanta 200 ESEM, at 10–20 kV, under low-vacuum conditions. The analysis of the root surface was conducted according to the Back-Scattered Electron (BSE) method and in X-ray microanalysis for energy dispersion (EDS).

**Figure 3:**
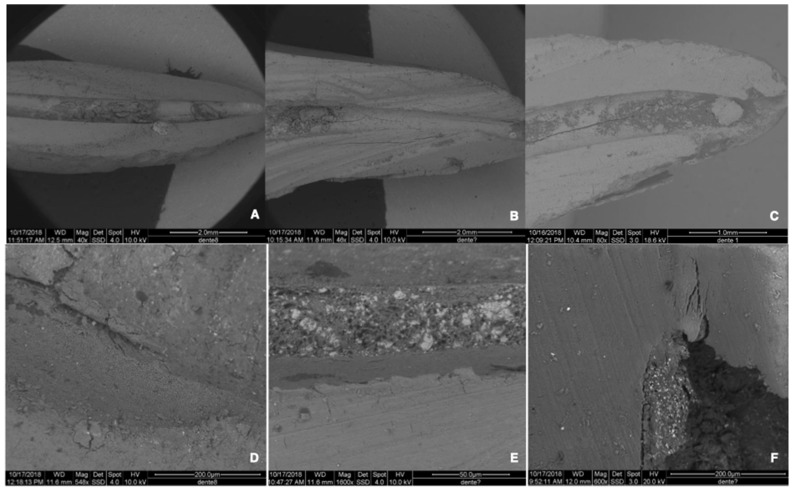
SEM images of the samples. (**A**–**C**) 50× magnification of BR, BR*, and AH group samples, respectively. (**D**–**F**) High-magnification images showing bonding at the interface between sealer and dentin, respectively, in BR, BR*, and AH groups. (**D**,**E**) Micrographs highlighting the *mineral infiltration zone* between CSBS and dentin.

Finally, two representative samples for each group were chosen, subjected to air drying, and examined at SEM (FEI Quanta 200 ESEM, CeSAR, University of Cagliari) at 10–20 kV and under low-vacuum conditions (*n* = 2 for each group) (Figure 2A,B). The analysis was performed using the Back-Scattered Electron (BSE) method and the X-ray microanalysis for energy dispersion (EDS). The magnification used varied from 30× to 1600× (Figure 3A–F). SEM observation showed the interfaces between sealer and dentin, and EDS analysis was used to identify the chemical composition of dentin, CSBS, and their interfaces (Figure 3D–F). 

#### 2.4.5. Data Presentation and Statistical Analysis

STATA/SE, version 14, software (StataCorp LP, College Station, TX, USA) was used for the statistical analysis. 

Of all data extrapolated from the evaluation of the samples (Table 1), the variables analyzed were: 1. percentage of residual filling materials in the whole surface of the canal (in %); 2. percentage of residual filling materials in the apical 3rd of the canal (in %); 3. times required for re-treatment procedure (in minutes); 4. re-establishing working length and patency (Table 2).

Kolmogorov–Smirnov test was used to check whether the variables had a normal distribution.

The variables showed a normal distribution. The experimental groups were compared using the ANOVA test and Student’s *t*-test with post hoc Bonferroni correction, where appropriate.

The significance level was set at *p* = 0.05. 

## 3. Results

Overall, the sample (36 teeth) presented the characteristics summarized in Table 1.

The variables were as follows: 1. percentage of residual filling materials in the whole surface of the canal (in %); 2. percentage of residual filling materials in the apical 3rd of the canal (in %); 3. time required for re-treatment procedure (in minutes) showing a normal distribution (Kolmogorov–Smirnov test *p*-value > 0.05). Consequently, these data were summarized as mean (SD) (Table 2). ANOVA analyses showed statistical relevance for RFM (%) (*p*-value = 0.038) and times required for the re-treatment procedure (min) (*p*-value = 0.003). Student’s *t*-tests with post hoc Bonferroni’s correction were applied to the variables where ANOVA was significant (Table 3).

### 3.1. Percentage of Residual Filling Materials in the Whole Surface of the Canal [RFM (%)]

All specimens of the three groups exhibited RFM (Figure 1 and Figure 3 and Table 1 and Table 4).

The lowest mean percentage of RFM was observed in BR (Table 4).

The most significant mean percentage of RFM was observed in BR* (Table 4).

The mean percentage of RFM was significantly different between BR and BR* (*p*-value = 0.048); the experimental group of teeth that were re-treated after one year showed higher values than those that re-treated after one month (Table 3).

### 3.2. Percentage of Residual Filling Materials in the Apical 3rd of the Canal (Apical 3rd RFM (%))

The mean percentages of RFM in the apical third are shown in Table 1.

No statistical difference was detected for this variable in the three groups. 

### 3.3. Time Required for Re-Treatment Procedure

The mean time to complete the re-treatment procedures was significantly different between the groups (Table 3). AH showed the lowest values compared to BR and BR* (*p* = 0.0001 and *p* = 0.0078). When BR and BR* were compared, there was no significant difference.

### 3.4. Re-Establishing Working Length and Patency

Patency and WL were re-established in all the teeth (100%) in the AH, BR, and BR* groups.

High-resolution SEM images showed RFM in the re-treated samples, confirmed the data obtained from the photomicrographs at SM, and revealed what could be considered an *infiltration zone* between CSBS and dentin (Figure 3A–F). 

**Figure 4:**
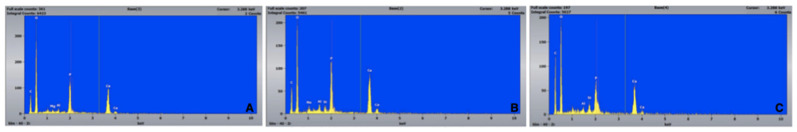
EDS microanalysis highlighted the presence of calcium phosphate ions within the dentin (**A**), *mineral infiltration zone* (**B**), and BioRoot RCS (**C**). More calcium phosphate ions were found in the *mineral infiltration zone* (**A**).

EDS microanalysis highlighted the presence of calcium phosphate in the *infiltration zone*, both in the re-treated samples previously filled with a CSBS and in the CSBS itself (Figure 4B,C). 

Finally, EDS revealed a more significant amount of calcium and phosphorus ions in the *infiltration zone* than in dentin and CSBS (Figure 4A–C).

## 4. Discussion

Failure after primary endodontic treatment can occur if infection persists in the root canal system. The root canal space should therefore be adequately cleaned and disinfected when performing a secondary treatment, by removing the previous filling, and re-establishing the WL. Furthermore, re-achieving patency in these cases significantly improves periapical healing rates [2]. 

Recently, there has been an increasing use of CSBS in endodontics [20,21]; however, the information available on the possibility of effectively removing these root-filling materials is not consistent. Hence, this study evaluated the potential of re-treating a relatively new bioactive sealer (BioRoot^TM^ RCS) compared to a more commonly used resin-based-sealer (AH Plus).

Most of the research performed on the removal of root canal sealers has used a storage time of 1 to 4 weeks [22,23,24,25,26], and the samples have been stored for a longer time before evaluation in only a few reports, which has never exceeded six months [27,28,29,30]. However, patients may require re-treatment several months or years after primary root canal treatment in a clinical setting. For this reason, part of the specimens filled with CSBS was stored for 1 month and part for 12 months before re-treatment in this experiment. To our knowledge, this is the first work that evaluates the removal of a CSBS following long-term storage. Interestingly, this study shows that the bioactive hydraulic sealer tested does not seem to exhibit washout 12 months following obturation, which may have particular clinical relevance.

To minimize the sampling bias, single-rooted mature human teeth with a completely formed root and a single straight and regular canal were selected (Figure 1), with strict instrumentation and irrigation protocol. This standardization allowed us to focus on the differences between the groups related to the characteristics of the sealers (BioRoot^TM^ RCS vs AH Plus), the time required to re-treat the CSBS (BR* vs. BR), and the possibility of regaining the working length (WL vs. WL-1).

To assess the percentage of RFM, various re-treatment and imaging methods have successfully been employed over time [19,31,32]. NiTi rotary instruments have been recommended to remove GP, and multiple studies have reported their efficacy, cleaning ability, and safety [31,33]. In this experiment, the removal of GP was performed first by means of Protaper Universal Re-Treatment, followed by leaving the samples in contact with a solvent for one minute. Re-treatment procedures were considered complete when no residual material could be seen on instruments at 4.5× magnification and further confirmed by intraoral periapical radiograph. Nevertheless, the observation of all samples revealed that the complete removal of filling materials was not achieved in any of the groups (Figure 3 and Table 3). This finding confirmed that the enlargement of the canal preparation, the achievement of the WL, and the lack of debris in the files could not guarantee the complete removal of the sealer from the canal walls. This observation is consistent with other reports [19,34,35] and should be considered during clinical re-treatment procedures [31]. 

Once the roots were split into two halves, root filling removal was photographed at the SM and evaluated through Image J software (Figure 2), a simple and efficient method to analyze a three-dimensional structure through a two-dimensional image. Further, to avoid biases, the third examiner randomly evaluated each image without knowing which treatment group it belonged to. Several studies assessed the amount of RFM following re-treatment by optical microscope inspection and digital evaluation of samples with ImageJ software [36,37,38]; hence, the method used in the present study is well established. In other reports, the removal of RFM was evaluated using high-resolution micro-computed tomography [19,31,34,39], an approach that allows the development of accurate three-dimensional models with a non-destructive imaging process [39], and enables the assessment of previous canal filling materials [40].

Our results show that obturation with the new bioactive hydraulic sealers does not permanently block the apical area, a finding that is in agreement with a previous report [41]. Using standardized straight canals made it possible to disclose the differences between the obturation techniques without the influence of the complexity of the tooth [19,34]. This can explain why previous studies evaluating more complex anatomies demonstrated a discrepancy with these results [42]. Furthermore, this study used the matched-taper, single-cone filling technique, which enalbes the easier penetration of rotary re-treatment instruments into the obturation [31]. While CSBS is hard upon setting, our study showed that apical patency and WL were achieved in 100% of the samples, indistinctively, whether the pilot or master cone was used. This finding confirms what was reported recently by Alsubait et al. [43].

Among the teeth obturated with BioRoot^TM^ RCS, the group re-treated after one year (BR*) showed a mean percentage of RFM significantly higher than BR (Table 3). The null hypothesis was thus rejected. This difference may be attributed to the increased mineral infiltration interface with time. It has been discussed that the precipitation of calcium phosphate ions within the dentinal tubules should explain the more effective sealing ability of the CSBS material over time [35,44,45,46]. 

According to the present results, the re-treatment time was affected by the type of sealer. This is in agreement with other studies [36,43]. Since there were differences in the time required for re-treating the samples, whether CSBS or RBS were used in our research, the null hypothesis was rejected. In our study, the AH group showed the lowest values. This finding is inconsistent with an earlier study [36]. However, a direct comparison cannot be made, due to the rotary instruments used for re-treatment. Finally, in our study, a long time was needed to complete the re-treatment procedures when the CSBS had been in the canal for a longer time, a condition justified by the observations mentioned above conducted via SEM. This difference was statistically significant; however, it may not have clinical significance. 

High-resolution SEM photomicrographs were also obtained to better observe the debris in the re-treated canals and to evaluate the cleanliness of the dentinal interfaces (Figure 3C,D). Two roots, representative of each group, were subjected to air-drying only, avoiding dehydration and potentially altering the samples. Thus, the images obtained were directly related to the scattering characteristics of the different materials analyzed (dentin, sealers, and gutta-percha, when present).

As previously reported [35], CSBS seem to induce mineralization at the interface with biological tissues, thus establishing a chemical bond between sealer and dentine, and SEM was used to observe this interface in our study. RBS exhibited continuity with the dentinal walls. In contrast, the interface CSBS/dentin appeared to be mediated by the formation of an *infiltration zone* (Figure 3D–F), which, on the basis of EDS microanalysis, was defined as a *mineral* *infiltration zone* for the presence of calcium phosphate ions (Figure 4A–C), which were also found in the CSBS. 

Regarding clinical safety, no instrument separation and procedural preparation errors occurred during the re-treatment procedure. This was probably related to the choice of working on easy samples. This aspect represents a limitation of our study, because it does not allow us to extend the results to curved canals. Another potential limitation is the solvent used to remove the root filling materials, even if its use was mostly chosen to help in cleaning every residual from gutta-percha, as there is no evidence that currently available solvents are effective on CSBS. We believe it may be necessary to develop a solvent that improves the re-treatment of CSBS.

## 5. Conclusions

The re-treatability of the novel BioRoot^TM^ RCS, based on this ex vivo model, can be considered manageable in teeth with simple anatomy. However, conventional re-treatment techniques are not always able to entirely remove it, especially when primary root canal treatment has been performed over a long time and a solid cementum-dentin sealing interface has been established. This may represent critical information for clinics, especially when re-treating long-term filled teeth with a CSBS. Still, these results need to be confirmed by studies performed in vivo.

## Figures and Tables

**Table 1:** Data of the entire study sample (*n* = 36).

Variables	Mean	Median	Standard Deviation	Min	Max	Range	IQR
RFM (%) *	29,104	28,900	9192	13,460	48,920	35,460	14,060
Apical 3rd RFM (%) **	18,025	14,355	13,759	0.540	68,230	67,690	20,730
*Times required for re-treatment procedure (min)*	2124	2075	0.865	1000	4520	3520	1000

* Percentage of residual filling materials in the whole surface of the canal. ** Percentage of residual filling materials in the apical 3rd of the canal.

**Table 2:** Summary values of the variables considered.

Variables	Mean	Standard Deviation
RFM (%) *	29,104	9192
Apical 3rd RFM (%) **	18,025	13,759
*Times required for re-treatment procedure (min)*	2124	0.865

* Percentage of residual filling materials concerning the area of the canal. ** Percentage of residual filling materials concerning the area of the apical 3rd of the canal.

**Table 3:** Student’s t-test with post hoc Bonferroni correction (variables where ANOVA was significant).

Dependent Variables	(I) Groups	(J) Groups	Mean Difference (I–J)	Sig.	Confidence Interval 95%	
					Inferior	Superior
RFM (%) *	AH, AH-1	BR, BR-1	7126	0.149	−1701	15,952
		BR*, BR*-1	−1775	1000	−10,601	7051
	BR, BR-1	AH, AH-1	−7126	0.149	−15,952	1701
		BR*, BR*-1	−8901	0.048	−17,727	−0.074
	BR*, BR*-1	AH, AH-1	1775	1000	−7051	10,601
		BR, BR-1	8901	0.048	0.074	17,727
*Times required for re-treatment procedure (min)*	AH, AH-1	BR, BR-1	−0.981	0.0001	−1397	−0.565
		BR*, BR*-1	−0.988	0.0078	−1687	−0.288
	BR, BR-1	AH, AH-1	0.981	0.0001	0,565	1397
		BR*, BR*-1	−0.007	0.9852	−0.741	0.728
	BR*, BR*-1	AH, AH-1	0.988	0.0078	0.288	1687
		BR, BR-1	0.007	0.9852	−0.728	0.741

* Percentage of residual filling materials in the whole surface of the canal.

**Table 4:** Mean and standard deviation of the percentage of remaining filling materials in the experimental groups and subgroups.

Variables	Overall	Subgroups (WL)	Subgroups (WL-1)
*BR*	23.76 (9.41)	22.37 (5.46)	33.09 (7.14)
BR*	32.66 (8.05)	32.24 (9.55)	25.15 (12.66)
*AH*	30.89 (8.19)	27.98 (10.04)	33.79 (5.13)

## Data Availability

Not applicable.
